# Supporting Parent Caregivers of Children with Life-Limiting Illness

**DOI:** 10.3390/children5070085

**Published:** 2018-06-26

**Authors:** Kendra D. Koch, Barbara L. Jones

**Affiliations:** Steve Hicks School of Social Work, University of Texas at Austin, 1925 San Jacinto Blvd., D3500, Austin, TX 78712, USA; barbarajones@mail.utexas.edu

**Keywords:** palliative care, special needs, parent, respite, life-limiting illness, caregiver, pediatric, psychosocial, stress, medically complex

## Abstract

The well-being of parents is essential to the well-being of children with life-limiting illness. Parents are vulnerable to a range of negative financial, physical, and psychosocial issues due to caregiving tasks and other stressors related to the illness of their child. Pediatric palliative care practitioners provide good care to children by supporting their parents in decision-making and difficult conversations, by managing pain and other symptoms in the ill child, and by addressing parent and family needs for care coordination, respite, bereavement, and social and emotional support. No matter the design or setting of a pediatric palliative care team, practitioners can seek to provide for parent needs by referral or intervention by the care team.

## 1. Introduction: Available and Needed Pediatric Palliative Care Services

Pediatric palliative care (PPC) programs typically help children and families with decision-making, communication, psychosocial support, pain and symptom management, and bereavement care [1]. However, as the population of children and families receiving pediatric palliative care services has grown to include those who have life-limiting, complex illnesses, recent research suggests that parents and families may need an expansion of these domains to include care coordination, respite, and education and support for medical complexity [2].

As developments in treatment and technology have led to prolonged life-spans for children with life-threatening, complex, chronic conditions, patients and families have expressed a need for support in the broadest definition of palliative care beyond end-of-life care [2,3,4,5]. Pediatric palliative care research and practice has begun to change the emphasis of palliative care articles from end-of-life topics to include more comprehensive topics that cover the span of a child’s life, from diagnosis to death, and all the life in between [2,6,7].

The American Academy of Pediatrics (AAP) recommends that patient- and family-centered care is an essential component of good pediatric palliative care practice [8]. Additionally, the International Meeting for Palliative Care in Children, Trento (IMPaCCT) standards developed by the 2006 consensus meeting of health professionals from Europe, Canada, the United States, and Lebanon adopted the stance of the World Health Organization (WHO) that “Palliative care for children is the active total care of the child’s body, mind and spirit, and also involves giving support to the family” [9]. In 2015, the professional consensus, “Standards for the Psychosocial Care of Children With Cancer and Their Families” [10] identified pediatric palliative care with an emphasis on care for the family, as one of its 15 essential standards of psychosocial care for children with cancer [11].

Despite consensus, and although increasing in number and scope, Pediatric Palliative Care (PPC) services internationally still do not meet the needs of many pediatric patients. International reviews of palliative care services find that even in countries with the most developed PPC programs, the vast number of children who might benefit never have access to PPC services [12,13,14,15].

While all countries have barriers to implementing adequate PPC services, lower- and middle-income countries are disproportionately challenged by lack of medical and financial resources, problems of access, lack of awareness of what PPC services offer, and lack of trained health care and social workers. Proponents of PPC have developed a framework to overcome barriers which includes, (1) working at all levels from health centers to governments to increase advocacy and awareness; (2) educating health care and social work professionals at different levels of expertise from a general approach to a specialist level of pediatric palliative care; (3) continuing to address disparity issues regarding access to medications; (4) implementing and evaluating a range of pediatric palliative care service models to meet differing resource, geographic, cultural, and disease-specific need; and (5) prioritizing pragmatic and translational research that acknowledges the need for culturally- and regionally-specific studies to provide medical and social best practices for providers [12].

Even in more integrated programs, referrals may be more likely to be made at the end-of-life and consider end-of-life concerns from the healthcare provider. Providers using a lifespan philosophy of palliative care would offer more holistic care that is closer to the time of a child’s diagnosis of a life-threatening illness [15]. This particular barrier to PPC underscores the need for healthcare providers to not only understand PPC best practices and philosophy, but also to continue to implement and refer to services that are holistic, comprehensive, and timely. Screening instruments may also be used by providers to standardize referrals, increasing the likelihood that patients and families will have the benefit of palliative care support [16].

Depending on the model of the pediatric palliative care service, healthcare professionals may not be able to directly provide needed services for families, however, PPC teams should be prepared to intervene or offer referrals for issues that further stress families, even if they do not seem to be covered by the umbrella of what PPC might typically address. In addition to more typical PPC services, the intent of PPC to prevent and relieve suffering can be best achieved when primary stressors for parent caregivers are addressed, including: (1) care coordination; (2) respite care; (3) peer and emotional support; (4) insurance and employment benefits; and (5) health and related supports [17]. Research outcomes for these five areas support (1) streamlining services and (2) minimizing the effects of caregiving burden. In addition, intervention research emphasizes healthy and intentional collaborations between healthcare professionals and families [17].

## 2. Parents as Caregivers

Understanding the types of caregiving that parents offer their children may help to guide the types of support a PPC professional might offer. Caregiving can be divided into five basic types of support: instrumental, personal, informational, medical, and emotional [18]. Instrumental, personal, and informational support are social supports. Instrumental caregiving, also known as instrumental activities of daily living (IADL) are those supports that allow a person (in this case a child) to live and engage in community. For example, going to school, driving (or being transported), communication with peers or teachers, or caring for a family pet. Instrumental activities are important to the social well-being of the child. Personal caregiving or personal activities of daily living (also known as habilitation) include those tasks of caregiving that are personal to the child: feeding, personal hygiene, managing incontinence, and dressing are in this category. Informational caregiving is generally managing information, medical or otherwise, for issues that need to be addressed by the person for whom the caregiver is caring. Although, this generally applies to geriatric populations. Medical decision-making, information on diagnosis, care coordination, and other information-specific exchanges might be the pediatric equivalent of this caregiving type. Emotional caregiving is what it seems—it is the care provided to the child to address emotional needs. In the world of children who receive PPC services, emotional care may reflect more specifically a parent’s need to address emotions associated with illness, such as sadness, hope, hopelessness, or fear. Finally, Medical supports are fulfilling those tasks associated with medical care, for example: changing a g-tube, tube feeding, suctioning, administering nebulizer treatments, managing a tracheostomy, adhering to a medicine regimen, positioning a child, or monitoring seizure activity [18]. Often overlooked is self-care. Self-care is a factor in caregiving because it promotes that caregivers also prioritize their own care. For many caregivers, this domain is the easiest to overlook. Primary caregivers may react with amusement or anger to suggestions that they care for themselves, because for them the word “care” implies not an affective state, but another task to be wedged in to a regimen of care that may already be full.

Parent caregivers of children with life-limiting illness are expected to assume many roles that extend across physical, emotional, social, and spiritual domains, including, everyday instrumental care provider, medical and financial decision-maker, advocate in education, patient advocate, nurse, relationship manager, care coordinator, communicator, transport service, insurance and financial support manager, and “typical parent” [19,20]. The level at which a care provider gives instrumental care may depend on the course or severity of the child’s disease, minimizing or increasing the need for help with tasks like hygiene, dressing, feeding, lifting, and transport [2,19]. A recent Italian study of 33 families who cared for children with life-limiting diseases showed parents spent an average of nine hours a day meeting medical needs [21].

Some tasks are more constant, requiring unaccounted for hours of mental time from caregivers. One of these—medical decision-making—begins at the point of birth and continues throughout the course of the child’s illness. The parent is the surrogate decision maker deciding on what treatments the child is to have, what medicines they will take, and which specialists they will see. This is the case whether the child is born healthy developing illness later, as in cancer or some neurodegenerative diseases, or if the child is born with congenital illness. Many parents express that decision-making adds to the burden of care for the child, but that it is an essential role of parenting [22,23]. In addition, complicated family dynamics or familial struggles, sleeplessness, financial or other stress, and pre-existing needs such as poverty may complicate care and decision-making [24]. The medical caregiving role is in addition to and sometimes at odds with the typical parental caregiving for the child because medical caregiving asks the parent to subject their child to difficult and sometimes painful procedures and experiences while the typical parental role is to protect the child from pain and discomfort [25].

Parent caregivers of children with life-limiting illness are vulnerable to negative social, psychological, physical, relational, individual, and financial sequelae. They are more likely to have depressive symptoms [26,27] and more likely to be fatigued, lack vitality, and have problems with sleep than are caregivers of typical children [19,28]. Parents of children with life-limiting illness suffer from social isolation due to caregiving tasks, threat of their child being exposed to pathogens, lack of respite care, and frequent hospitalizations [29]. They are also more likely than parents of typical children to encounter financial problems from healthcare costs and the need to reduce work to meet caregiving demands [19,26,30].

Each family constructs care for their ill child in a way that is unique to their family structure, strengths, and challenges. Caregiving may be both indirect and direct (instrumental), and parents who offer more direct care to the child are at higher risk of personal negative outcomes [26]. Because 90% of primary caregivers of children with chronic or complex needs are mothers [26], it makes sense that mothers of these children, more than fathers, are more often affected by anxiety, depression, pain, and physical health symptoms [31,32,33]. Pediatric Palliative Care (PPC) standards include caring for both the ill child and their family [1].

Care for the parent in pediatric palliative care has been justified by the ethical imperative of caring for the whole family in pediatric palliative care [34,35]. Although the impact of a child’s life-limiting illness on parents is well articulated in the literature [36], providing services that are supportive of parents is sometimes still not well executed, perhaps in practice still being treated as an ancillary or optional part of pediatric palliative care practice [3]. As the child’s primary caregivers, parents are a direct and highly influential factor in child wellness and health outcomes, and caring for the parents is essential to caring for the child [1,35].

Ethics and professionally agreed upon consensus statements have provided compelling reasons for professionals to care for families, including parent caregivers. To reinforce this foundation, in addition to offering practical direction for healthcare professionals to provide the best support to parents, this article will articulate how care for the parent directly impacts the ill child. By offering a brief overview of supportive research, this article underscores that caring for the parents of children with life-limiting illness is more than preferred, it is evidence-based and essential and ultimately leads to better outcomes for the ill child.

## 3. Care for the Parent Directly Impacts the Child

The most pragmatic justification for directly assessing and addressing parental need is in the understanding of the parent-child connection, which is a continual interaction of cognitive, behavioral, and emotional factors designed to protect the child [37]. Because of this connection, children with life-limiting illness may be directly affected by the psychological well-being of their parents. For example, a parent’s response to their child’s pain may affect the child’s experience of that pain [38]. In one study, children whose parents were oriented toward their pain and distress, without being self-oriented (responding out of self-protection to the distress that the child’s pain may have caused in the parent) had less observable pain and distress and a more positive disposition during cancer treatments [39]. Similarly, in children with chronic pain, parents who engaged in catastrophic thinking about pain, had children who engaged in greater catastrophizing of pain [40].

Evidence suggests that parents facing consistent and premorbid stressors may experience a neurologic process dominated by the limbic distress response, instead of a more cognitive response of adaptive coping. This means that parents who are already under strain will become more behaviorally distressed in crisis situations, instead of accessing more adaptive and resilient responses [38,41]. As shown previously, this distress response may increase a mirrored response in the child, affecting both emotional and physical outcomes for children with life-limiting illness. Research also suggests that depression among caregivers may lead to difficulty communicating with providers and less satisfaction with medical care [42,43]. It is not only poor mental health that affects children, parent well-being is also associated with increased well-being in siblings of ill children. Studies of posttraumatic growth (positive changes experienced in the face of adversity) show that higher maternal posttraumatic growth levels are associated with fewer behavior problems in siblings [44].

Given the evidence of such strong associations between parent well-being and child well-being, healthcare providers should offer care to parents, not only because it is compassionate, not only because parents have an ethical claim to care, not only to involve parents in decision making and medical education, and not only because including parents in the care schema acknowledges the context in which the child lives, but also because the well-being of parents, who often provide the most instrumental, daily care for the ill child [32,45], directly affects outcomes for the ill child [32,45].

## 4. What Providers Can Do

### 4.1. Assessing Parents

The importance of adequate and ongoing assessment that includes assessing the psychosocial needs of the child and their parent caregivers has been established by the Institute of Medicine, the American Cancer Society, the National Comprehensive Cancer Network, the Psychosocial Standards of Care Project for Childhood Cancer (PSCPCC), and the Association of Pediatric Oncology Social Workers [10,36,46,47,48,49]. Initial consensus and literature on the topic of parent caregiver assessment (or screening, which is a less comprehensive approach to identifying problems or needs) began in pediatric cancer [36]. More recently, healthcare providers and researchers have observed the increasing number of medically complex children outside of cancer and more typically progressive diseases, and have suggested that both practice and research focus on increasing assessment and evidence-based practice in that population of children and families as well [2,17].

However, although healthcare professionals are aware of the increased physical, emotional, personal, financial, and relational impact on parent caregivers, assessing their distress in these areas is still not standard practice [45]. Four common barriers to professional assessment of parent needs include: (1) inadequate staff funding that leads to lack of time to address clinical needs; (2) staff inexperience with parent/adult engagement, coupled with lack of training on parent engagement; (3) pediatric institutions having an unwillingness to accept parents, who are seen as “adult patients”; and (4) lack of understanding as to how to bill and document parent experience/treatment in the context of pediatric specialist practice [11]. These barriers create large gaps in care, evidenced by a recent study of Children’s Oncology Group Institutions, which reported that a meager 9% of institutions used empirically supported psychosocial evaluations, and further, that only 50% of parents received assessments or psychosocial support within the first 30 days of their child’s diagnosis [50]. In some cases, there is a need for increased social work and other psychosocial support staffing to meet the needs of both the child and parent.

While some assessment instruments do exist in the domains of caregiver burden, satisfaction with healthcare delivery, caregiver needs, caregiver quality of life, and caregiver distress [45], they are not always specific to parental distress. Additionally, it is difficult for providers to know if instruments are intended for clinical or research purposes [45]. In a recent review of instruments, researchers found 59 instruments that might be useful for assessment of parents of children with chronic or complex illness, however, of those, only 12 were found to be reliable, valid, self-reported, and minimally burdensome (having fewer than 20 items) [45]. Further, research suggests that even when assessments occur, often the interventions that follow may not be evidence-based. In the multi-site study referenced previously, only 11% of the subsequent interventions (post-assessment), were empirically supported [50].

While parental distress may be assessed in pediatric life-limiting illness, that assessment is still not systematic [10,36]. To ameliorate symptoms and support coping, ideally, parental distress screening and assessment would be routine in all pediatric palliative care settings, but this would require adequate staffing, specific and tailored measures, as well as an increased understanding of the direct impact on the child of parental distress and appropriate interventions to effectively lessen that impact [27,36,51].

### 4.2. Pain and Physical Distress

Parents report that witnessing physical distress and pain in their children is extremely troubling. Parental well-being is closely tied to the needs and suffering of the ill child [52]. Managing pain and physical distress in the child is the most direct way to meet immediate needs for the child and for their parent. Easing symptoms of physical distress and pain in the patient may have the added benefit of reducing psychological distress in the patient and vice versa [38]. Parents often cite pain management as being the aspect of pediatric palliative care that is most important to them [53,54]. Each disease process presents different physical challenges to pediatric palliative care professionals, who broadly assess for symptoms of physical distress including pain, respiratory symptoms of dyspnea or “air hunger,” fatigue, spasticity, gastrointestinal problems including constipation and motility issues, issues of positioning, and chronic irritability, especially in neurologically impaired children [55,56].

### 4.3. Communication

Second only to pain and symptom control, parents express that good communication is the most highly prioritized aspect of pediatric palliative care [57,58,59]. Compassionate and effective communication has the potential to foster trusting relationships, provide anticipatory guidance, offer information, support emotions, manage uncertainty, assist in decision-making, and enable patient and family self-management [35,55,60,61].

Throughout their child’s life, parents of children with life-threatening illness are asked to understand masses of information, assimilate difficult news, and make decisions based on communication with their child’s healthcare team. Parents want to be able to hear information in a manner that allows them to trust the content that they are receiving from their child’s team. Parental trust is increased when information and discussion is presented in ways that are accessible and helpful to parents. Communication is made of content (*what* is being communicated) and manner (*how* the content is being communicated). The manner of communication includes the tone, language, style, and cultural sensitivity with which the content is conveyed [61]. Professionals may attend to both the what and the how by focusing on these areas: timeliness (of test results, labs), language (using credentialed interpreters if needed), style (i.e., directive versus non-directive), and intricacies in communication like respect (calling an infant by name), or cultural humility (avoiding the use of culturally-bound metaphors and acknowledging cultural norms of patient and family) [61].

Each family will have specific preferences for the style of communication and amount of information they would prefer to receive. Some parents want limited information, finding certain types of information upsetting or reducing hopefulness. However, most parents express that they want more medical information [62] about their child’s illness, not less [63,64]. For these parents, information may help them to cope and regain a sense of control, reducing the uncertainty of the situation. Still, it is important to work with the family to understand their preferred communication style and timing. Healthcare professionals can ask parents, “How would you like information shared? All at once? A little at a time?” “Who needs to be here when we communicate?” “How should we talk with your child?” [55,61,65].

Anticipatory guidance relies on communication to describe future symptoms or conditions that may develop as part of the child’s illness. Depending on the illness and its trajectory, the content of each guiding conversation is different. For instance, parents of a non-ambulatory child with static encephalopathy who is at far greater risk of developing recurrent aspiration pneumonia will require different anticipatory guidance than parents whose child has cancer with a poor prognosis [66]. Information regarding potential risks, probable outcome, and choices for treatment are all areas to be explored. In the instance of a child with static encephalopathy, the parents might be told that their child is at risk of recurrent infection from aspiration, opening the discussion to the parents’ wishes for future use of medical technology and their values and goals [61]. PPC practitioners understand that communication is often not one conversation, but many. As with all of pediatric palliative care, anticipatory guidance is more than a medical issue, it encompasses social-emotional and spiritual domains of care as well; acknowledging this, Klick and Hauer (2010) offer brief phrases that help PPC to assess for needs and guide discussions with parents and patients, “Who do you use for support?” “Are you able to do the things that you enjoy doing?” “What are the challenges getting through each day?” and, “Do you have a faith or spiritual belief that brings you support?” [67].

Practitioner attunement to the child and family is a critical component of PPC and can enhance delivery of PPC services. Davies et al. recommend that healthcare practitioners attune to the patient and family’s situation using six techniques: (1) *orienting* to all of the observable factors present in a conversation, from the state of mind of the practitioner, to the situation of the patient and family; (2) *seeking parents’ perspectives* by providing space and asking questions such as, “What do you understand about the illness?” “What supports do you have?” or “What is the thing that you are most afraid of?” (3) *discerning* by observing and listening to parent responses and nonverbal cues to determine how best to approach the conversation and what is most important to them; (4) *shaping* a thoughtful response that considers the parents’ states of mind and takes the most salient concerns of the parents to plan direct care activities; (5) *checking* by evaluating the effectiveness of an intervention, or by following up on promises to check back later or find out more information for parents; and (6) *reflecting* by purposefully and objectively considering their interactions with patients and families, their responses, attitudes, worldview, and behavior, noting opportunities to better attune and to find meaning in their experiences [68].

Communication must remain an ongoing and dialogical process that acknowledges that a parents’ way of thinking about their child’s care may change over time. End-of-life and care transition conversations especially are directly related to the child’s health status, symptoms, and quality of life, thus requiring healthcare professionals to reassess the health status of their patient, the needs of the family, and the type of communication needed to address each. Although different in content, each of these conversations require that the PPC clinician uses a team approach, enlisting interdisciplinary expertise to exchange information, promote anticipatory guidance, respond to child- and- family emotions, make decisions (including managing uncertainty and decisional regret), and enable patient and family self-management.

### 4.4. Decision Making

Decision-making support is the backbone of pediatric palliative care services. Historically, PPC has focused on end-of-life decision-making. Although this trend is changing, often research about parent decision-making for children with life-limiting illness is still overwhelmingly focused on end-of-life. As the field continues to grow and children with life-limiting illness continue to live longer, healthcare professionals can acknowledge the need for continued assessment, treatment, and referral to address the needs of parents of patients who may have illnesses that span decades. These parents engage with decision-making (DM) that often includes considering mundane, everyday questions not related to end-of-life conversations or processing a new illness status or diagnosis.

Practitioners may support parental decision making by considering these factors when guiding parents: (1) the complex and different roles that clinicians and parents have in the decision making process; (2) the parent’s changing understanding of the child as someone with a future and on whom now-unmet expectations have been placed; (3) that diagnosis of a life-limiting or life-threatening condition is an assault on the life of the family; (4) that for the sake of the family and preserving and maintaining normalcy, parents tend to push against the intrusion of the disease in everyday life; (5) that an individual’s and parent’s view of illness changes over time; (6) that parents use information in ways that clinician’s may not expect; and (7) that parent’s and clinicians may view the child differently [69].

Parents generally desire to be involved in decision-making for their children [63,70,71,72,73,74,75,76,77]. This is not changed by a parents’ experiencing decisional regret or guilt, in fact, parents may experience both whether they were the primary decision-maker or not [72,78]. There are, of course, exceptions to this—rarely, parents prefer that the physician or medical team be the decision-makers [78,79,80].

Parents view their child-focused decision making as part of “being a good parent” [63,81]. Their self-concept as expert, advocate, and protector for the child make their involvement in DM imperative [76,82]. Despite developments in decision-making ethic that promotes patient and parent autonomy as the primary concepts in decision-making practice, parents report barriers to implementation of decision-making that values their choices. Findings suggest that healthcare professionals (especially, physicians) hold values and goals for the ill child that may be at odds or discordant to those held by parents [62,83]. Further, parents sometimes felt that they had not been involved in even life-and-death decisions at all [78,79,80,82]. In some circumstances, parents felt like they were involved, but given such limited information or choice, that they had only one choice, or the decision was all-but-made for them [22,78,79,84]. Although, at least in principle, medical ethic and discourse have embraced autonomous choice, most parents actually prefer some level of shared or collaborative decision making. They want to be able to talk with healthcare providers and make a decision with them [71,72,85,86,87,88].

Whatever the information conveyed from the PPC practitioner, parents need and want information that allows them to make the decisions they feel will be best for the child [63]. Even if it is unpleasant, parents prefer the truth. Parents want the truth presented in ways that they can understand and that “leave room” for hope [64,89,90]. Truth telling is not only the preference of parents, it is a moral imperative. At times when telling the truth is difficult, PPC practitioners can consult with team members for support and remember that telling parents the truth gives them the information that they need to make the best decisions they can for their ill child [64,89]. Practitioners should also remember that they are part of a team and draw on the strengths of the team to support themselves and the family, conveying to parents that, “no matter what comes next, we will be here for you and your child” [35].

Healthcare professionals and families alike may find decision-making (DM) tools (or “Decision Aids,” DA) such as the “Ottawa Family Decision Guide” [91] or the “Caring for Health: Child Tracheostomy Decision Guide” [92] to be helpful when making a decision that can be aided by a benefits and burdens paradigm. These DM tools may allow parents and their healthcare team to consider questions in a systematic way that leads to a broader and richer discussion between the PPC team and parents.

### 4.5. Care Coordination

Although most often associated with primary care or medical home models, care coordination should exist wherever pediatric patients receive care. The vulnerability of children, their “developmental trajectory, dependency on adults, differential epidemiology of chronic disease, demographic patterns of poverty and diversity, and overall dollars” heightens their need for well-coordinated care [93]. A recent study of 735 parents with medically complex children ranked care coordination as one of the top two most challenging areas for parent caregivers [94]. The greatest challenges to care coordination in complex pediatric populations is in poor communication between services and providers [95].

Because PPC teams differ in processes, roles represented, and settings, it is difficult to prescribe one particular method to address care coordination in practice. However, Klick and Hauer suggest that the primary objectives for PPC practitioners addressing care coordination might be (1) collaborating with specialists; (2) identifying resources and partnering with community programs; (3) identifying financial resources and payment mechanisms; and (4) partnering with school programs [67]. The second and third items assume that adequate screening and assessment have been completed to inform the practitioner of the needs and challenges faced by the patient and family [36]. Care coordination is as unique to each family as communication and other palliative care tasks. It should not be limited to providing the same list of generic referrals or general suggestions to every family.

The care coordinator within a PPC team may be filled successfully by any number disciplines, although social worker, nurse, or nurse practitioner are the most commonly represented [96,97,98]. PPC teams may review resources by considering what types of patients need the most care coordination, and at what times. PPC teams may anticipate care coordination needs by reviewing the characteristics of their population of patients with the understanding that different populations of patients often have different types of needs. One study found shorter but more intensive needs for patients with malignant disease, when compared to patients with non-malignant disease who needed more hours of management and coordination overall, but spread over a much longer period of time [99]. Whatever their discipline, the presence of care coordinators is a particular support to parent caregivers and has been associated with reduced parental stress and increased caregiver satisfaction [94,100,101].

### 4.6. Respite Care

Parents of children with life-threatening illness need occasional respite from caregiving [26]. Even if it is only a few hours at a time, breaks from direct (instrumental) care help increase parents’ quality of life (QOL) and stem burnout, including symptoms of fatigue, psychological adjustment, depression, and anxiety in parent caregivers [102,103]. Children with PPC needs suffer from a range of diagnoses which present varied trajectories of illness, even within the same diagnosis. The unpredictability of these trajectories means that parent caregivers need consistent respite care on which they can rely. Access to respite varies with family resources. Some government programs pay for respite services, some do not. Some families have larger groups of family or friends from which to pull. Some diagnoses are easier to manage without nursing care or medical knowledge. Parents do report that often it is difficult to find trustworthy respite workers and that having respite at the cost of not knowing or fully trusting the worker who is with your ill child is worse than having no respite at all [104].

PPC professionals should ask about parental needs for respite, before parents show signs of burnout, exhaustion, or fatigue. Small and consistent doses of rest throughout the trajectory of illness allow parents to process and adjust in small increments, instead of trying to recover from physical and emotional exhaustion. If parents are open to finding respite resources, PPC teams should have resources and referral information ready to give to parents and should help parents access resources, if needed [105].

### 4.7. Social and Emotional Support

Parents of children with life-threatening illness have a range of social and emotional needs stemming from an array of feelings, emotional overwhelm, and high levels of stress. The emotional outcomes found in research literature include anxiety and depressive symptoms, guilt, stress, fear, varying degrees of uncertainty and disbelief, denial, powerlessness, anger, sadness, and anticipatory and realized grief [11,21,105].

Psychological interventions aimed at parental distress in PPC cancer settings are still emerging, with studies that are limited by small numbers and lack of appropriate controls [11]. However, among reviewed interventions, several do stand out as offering hopeful outcomes: Problem Solving Skills Training (PSST) has been shown to be effective in reducing negative affect in mothers of children newly diagnosed with cancer [11]. Progressive Muscle Relaxation and Guided Imagery Techniques have both been shown to reduce anxiety and improve mood in parents of children with cancer [106]. When it is available, families are open to information that helps them lessen their own psychological and emotional concerns [107]. Interventions that offer psychoeducation and promote the well-being of the caregiver have some protective effect in limiting increases in distress [17]. These interventions included both face-to-face check-ins as well as interventions that used phone calls, with no face-to-face engagement [17].

Because a lack of social support has been associated with higher levels of distress, psychological morbidity, and post-traumatic stress disorder (PTSD) [108,109], and because increased community and peer social support have been shown to ameliorate distress in parents, it may be beneficial for PPC practitioners to facilitate channels of personal and systems engagement between parents of ill children and community organizations or peer support [108].

Research shows that caregivers’ growth in relationships with others during difficult times is likely to have effects on family members, the ill child, and on the caregiver [44,110]. Relationship-focused coping strategies may be helpful to maintain and build relationships during periods of stress. Using these strategies, parents can be encouraged to consider the responses of others involved in care, while being given the reassurance that both similar and dissimilar (complementary) coping styles may exist and be helpful in parent dyads [111]. Relationship-focused coping includes activities such as, putting yourself “in someone else’s shoes,” active listening, trying to understand how someone else feels, finding equitable solutions, displays of positive feelings, and promoting empathy in relationships. These activities allow caregivers to nurture and sustain relationships, while reducing threat and defensiveness in stressful times [112]. Because of pre-existing socio-ecological issues, PPC services alone may be unable to address the social and emotional needs of parents [108]. However, it has been suggested that it is not necessarily an “intervention” that decreases distress for caregivers, but the practice of good pediatric palliative care that includes the previously-mentioned treatment of symptoms (in the patient), good communication, care coordination, and decision-making support that affect psychological outcomes for parents in PPC settings [108]. By practicing optimal PPC, practitioners help to decrease the likelihood of further deterioration in the parent and decrease the likelihood of further parental stress from poor communication, uncertainty or regret in decision-making, or distress from witnessing the suffering of a child.

## 5. Modeling Self Care through Reflective Practice

In order to offer the most compassionate care to parental caregivers, healthcare practitioners must engage in reflective practice, acknowledge that providing care to children with life-limiting conditions and their families is emotionally taxing, and practice intentional self-care and self-compassion. Caring for children who are suffering and their families can naturally lead to a sense of personal struggle and distress often referred to as burnout, compassion fatigue, or secondary traumatic stress [113]. Moral distress can occur for practitioners when they witness patient suffering and when they cannot alleviate that suffering. Moral distress is more common for practitioners delivering direct patient care in acute situations and can be a leading cause of burnout and staff turnover [114].

While it is commonly understood that caring for those who are suffering can lead to distress, how to prevent this is less studied [113]. Self-care and self-compassion are critical skills for the pediatric palliative care practitioner. Strategies that can assist include focusing on work-life balance, identifying a sense of meaning, and developing personal skills that help manage the stress [113]. It is expected that pediatric palliative care practitioners will experience struggle, but the key to managing this is to first engage in reflective practice. This self-reflection and self-care is not only preventative for the practitioners but can serve as a model for family caregivers about the critical importance of self-compassion and care. Institutional solutions in pediatric palliative care that can alleviate suffering include education about compassion fatigue and moral distress, on-site support, debriefing and support groups, mentorship, high functioning interdisciplinary teams, adequate staffing, bereavement, and memorials [113,115]. A recent study found that it can be helpful when pediatric palliative care teams are able to offer each other respect, nonjudgmental validation, and open communication [116]. Reflective practice is important not only to PPC providers, but also to parents. By watching PPC practitioners take time and space for themselves, parents may be inspired to seek out opportunities that promote wellness and caring for self, as well.

## 6. Bereavement

Sometimes bereavement is confused with grief. Grief is “primarily the emotional (affective) reaction to the loss of a loved one through death. It is a normal, natural reaction to loss” [117], while bereavement is a “broad term that encompasses the entire experience of family members and friends in the anticipation, death and subsequent adjustment to living following the death of a loved one” [118]. Parents who have lost their child to death experience a number of symptoms that, although negative, may be normal parts of bereavement, including: depression, anxiety, grief, guilt, and/or existential or spiritual distress [119,120]. These symptoms may persist over long periods of time [119], and affected mothers are at risk for poorer bereavement outcomes [121].

PPC professionals understand the need for services that address the psychosocial and emotional domains of bereavement, but PPC professionals should remember that bereavement also includes “the adjustment to living” after the death of a loved one. This adjustment may also include changes in health, relationships, and finances [122,123,124,125,126]. After a simple screening, PPC professionals should be prepared to advise and refer parents to appropriate services to receive further assessment and to address needs in these areas [127]. Instruments such as the Bereavement Risk Index or the Prolonged Grief Inventory may be used to assess for grieving that may need intervention or referral from the PPC team to more appropriate, long-term professional help.

Parents and other bereaved family members may grieve for years without complication, but for a subset of parents and other family members the grief can be unrelenting and problematic. Signs of post-traumatic stress disorder (PTSD) (i.e., intrusive memories, avoidance of reminders, negative alterations in cognitions and mood, and marked alterations in arousal and reactivity) [128], or grief that does not subside in intensity and focus over time should alert healthcare professionals that mental health intervention is needed [129,130].

In general, parents indicate that they want and appreciate follow-up by their child’s healthcare team during bereavement [131]. The “Standards for the Psychosocial Care of Children with Cancer and Their Families” proposes that “A member of the healthcare team should contact the family after a child’s death to assess family needs, to identify those at risk for negative psychosocial sequelae, to continue care, and to provide resources for bereavement support” [131].

There is very little research currently available demonstrating effectiveness of intervention measures for bereaved parents and siblings. A recent review found of 129 studies retrieved for full screening, only eight were rigorous and comparative studies. More well-designed randomized controlled trials are needed to present practitioners with effective interventions for bereaved parents [132].

## 7. Conclusions

Optimal pediatric palliative care includes care for both the child and the family. The well-being of children depends in large part on their parents’ well-being. The presence and involvement of parents in every aspect of their child’s care is essential to good care. The role of parents as primary caregivers to the ill child means that parents and practitioners are partners in decisions of care, and that information about the ill child should be provided for the parents and for the ill child. Therefore, care for parents can reduce the distress of both the parental caregiver and the child with life limiting illness. Even if it is in referral, PPC should meet the needs of its expanding complex chronic illness population by more broadly assessing for/meeting family needs. Parents are a critical component of a child’s well-being and the PPC interdisciplinary team should strive to provide routine psychosocial assessment, evidence-based interventions, shared decision-making, organized respite, and attention to distress for all parental caregivers. In so doing, PPC meets its goals of caring for the child in their primary context—in their family.
